# A preliminary computational outputs versus experimental results: Application of sTRAP, a biophysical tool for the analysis of SNPs of transcription factor‐binding sites

**DOI:** 10.1002/mgg3.1219

**Published:** 2020-03-10

**Authors:** Shirin Moradifard, Reza Saghiri, Parastoo Ehsani, Fatemeh Mirkhani, Mina Ebrahimi‐Rad

**Affiliations:** ^1^ Biochemistry Department Pasteur Institute of Iran Tehran Iran; ^2^ Molecular Biology Department Pasteur Institute of Iran Tehran Iran

**Keywords:** EMSA, SNP, sTRAP, transcription factor, transcription factor‐binding site

## Abstract

**Background:**

In the human genome, the transcription factors (TFs) and transcription factor‐binding sites (TFBSs) network has a great regulatory function in the biological pathways. Such crosstalk might be affected by the single‐nucleotide polymorphisms (SNPs), which could create or disrupt a TFBS, leading to either a disease or a phenotypic defect. Many computational resources have been introduced to predict the TFs binding variations due to SNPs inside TFBSs, sTRAP being one of them.

**Methods:**

A literature review was performed and the experimental data for 18 TFBSs located in 12 genes was provided. The sequences of TFBS motifs were extracted using two different strategies; in the size similar with synthetic target sites used in the experimental techniques, and with 60 bp upstream and downstream of the SNPs. The sTRAP (http://trap.molgen.mpg.de/cgi-bin/trap_two_seq_form.cgi) was applied to compute the binding affinity scores of their cognate TFs in the context of reference and mutant sequences of TFBSs. The alternative bioinformatics model used in this study was regulatory analysis of variation in enhancers (RAVEN; http://www.cisreg.ca/cgi-bin/RAVEN/a). The bioinformatics outputs of our study were compared with experimental data, electrophoretic mobility shift assay (EMSA).

**Results:**

In 6 out of 18 TFBSs in the following genes *COL1A1*,* Hb ḉᴪ*,* TF*,* FIX*,* MBL2*,* NOS2A*, the outputs of sTRAP were inconsistent with the results of EMSA. Furthermore, no p value of the difference between the two scores of binding affinity under the wild and mutant conditions of TFBSs was presented. Nor, were any criteria for preference or selection of any of the measurements of different matrices used for the same analysis.

**Conclusion:**

Our preliminary study indicated some paradoxical results between sTRAP and experimental data. However, to link the data of sTRAP to the biological functions, its optimization via experimental procedures with the integration of expanded data and applying several other bioinformatics tools might be required.

## Graphical abstract

We tested sTRAP to do some analysis on DNA variations in the cognate binding sites of specific transcription factors, for which there were the results of the experimental approaches, in the literature. At the same time, we compared the results of both of the procedures with the data of another bioinformatics model RAVEN, as an alternative one.

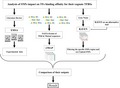

## 1 INTRODUCTION

In recent years, the increasing access to high‐throughput data of sequencing have explained the pathology of several diseases by the analysis of variations in the noncoding regions of the genome, transcription factors‐binding sites (TFBSs) being among them (ENCODE Project Consortium, 2012; MacArthur et al., 2017; Maurano, Wang, Wang, Kutyavin, & Stamatoyannopoulos, 2012).

The specific binding of transcription factors (TFs) to their target‐binding sites is a critical component of gene regulation at transcription and expression levels, a hallmark of several biological processes, including development, differentiation, and evolution, to name a few (Lai et al., 2019; Lambert et al., 2018; Savinkova et al., 2013). Several single‐nucleotide polymorphisms (SNPs), affecting TFBSs might be potentially involved in either destruction or creation of the new TFBSs, resulting in a genetic disease or a phenotypic trait (Chorley et al., 2008; Kumar, Ambrosini, Ambrosini, & Bucher, 2016; Rana, Coshic, Coshic, Goswami, & Tyagi, 2017).

The binding difference of a TF for the reference and alternate alleles might be linked to the emergence of the diseases. So, access to a bioinformatics tool with a capacity of such prediction would be very valuable in creating the related hypothesis on the issue. However, it is a challenging task in the functional genomic analysis. Previous efforts have proposed several computational models and tools to compute the impacts of the SNPs on the binding affinity of the TFs; however, due to the shortness and degenerateness of TFBSs, some of approaches were found to be impractical (Boyle et al., 2012; Chowdhary et al., 2012; Mathelier & Wasserman, 2013; Riva, 2012).

Manke et al. introduced a new biophysical model dedicated to predict the impacts of SNPs of the target TFBSs on their related TFs binding affinities (Manke, Heinig, Heinig, & Vingron, 2010). The authors represented the sTRAP web tool with the potential capability to compare the wild and mutant motifs of TFBSs in interaction with their cognate TFs and to quantify the difference between binding activity scores of TF for the allelic sequences. The tool is sequence based and takes advantage of the application of position weight matrices (PWMs), frequently used model, to compute TF–TFBS‐specific interaction (Zhao, Granas, & Stormo, 2009; Zhao, Ruan, Pandey, & Stormo, 2012) and the fixed‐length TFBS models for such prediction.

The present study addresses the analysis of the binding affinity variations of putative TFs due to SNPs introduced in their TFBSs. The study's objective was to check for compliance between the data predicted by sTRAP and those of experimental approaches in the literature.

For any model (biophysical or bioinformatics) to become a predictive tool, some validation against wet‐lab data are required (Cooper et al., 2018). If the analysis is properly conducted with limiting measurement uncertainties, the model would be capable and functional in a true prediction. Otherwise, something might be missing in the model, which should be introduced in its structure. However, the experimental procedures are not the exceptions of this rule. They also need to be validated by other approaches (Cooper et al., 2018). The challenge of the biophysical model versus the experimental approach might bring two important kinds of outcomes; the high compatibility or a great contradiction between their outputs. The results of both circumstances would be of worth to be reported because the knowledge on the power or inabilities of the model might enable the user to design his project and get an accurate conclusion.

## 2 METHODS

### 2.1 Data collection

In this study, a literature review was done to provide the experimental data by collecting the eligible studies with the required information, relevant to our commitment. Those investigated the impact of the SNPs in TFBSs on the binding affinities of related TFs, by experimental approaches such as EMSA, were selected for preliminary analysis. Then among them, the articles focused on the nuclear extract or cell extract as the source of TFs for their analysis were excluded from our study. While, the data of the articles with the application of recombinant or synthetic TFs were included in our implementation. The articles of Mann et al. (2001) and Savinkova et al. (2013), among the eligible studies, obtained by literature review, were the only sources for extraction of the candidate TFBSs motifs for our analysis. They reported the functional analysis of Sp1 and TBP (TATA‐Binding Protein/TATA Box) TFs binding affinity by using electrophoretic mobility shift assay (EMSA) technique, recombinant TFs, and synthetic DNA target sites, respectively. The latter group also found that the experimental results of TBP/TATA, highly correlated with those predicted by in silico prediction approach based on PWMs, they used in their study (*r* = .822, *α *< 10^–7^).

### 2.2 TF affinity analysis

The bioinformatics analysis was performed using the wild and mutant DNA sequences of the selected TFBS motifs from the sources mentioned above. sTRAP (http://trap.molgen.mpg.de/cgi-bin/trap_two_seq_form.cgi; Thomas‐Chollier et al., 2011), the computational and biophysical tool, was applied to evaluate the DNA motifs to assess the impact of SNPs in the TFBS on TF‐binding affinity. The used input consisted of the DNA sequences in FASTA format (Thomas‐Chollier et al., 2011), the length of the motifs in the bioinformatics analysis was considered to be as long as the synthetic target sites used in the experimental techniques, to avoid any deviation in the predictions. In the other strategy, 60 bp upstream and downstream of the SNPs were included in the evaluation. In the motif analysis, the highest score among those obtained by using different matrices, was considered as the related binding energy.

### 2.3 Analysis with RAVEN

Also, we utilized the regulatory analysis of variation in enhancers (RAVEN) (http://www.cisreg.ca/cgi-bin/RAVEN/a; Manke et al., 2010) as an alternative tool, due to its application together with sTRAP by Thomas‐Chollier et al. (2011) in their study.

## 3 RESULTS

### 3.1 The outputs of bioinformatics versus experimental data

The 18 TFBSs with SNPs (located in 12 genes), experimentally analyzed by the other researchers, were included in our study and scored against the wild TFBSs, using bioinformatics tools; sTRAP and RAVEN. One out of 18 (in *COL1A1* gene), was the target for the Sp1 transcription factor although, the remained 11 cases were those being the cognate binding sites for TBP transcription factor, inside several genes (Table 1).

**Table 1:** Functional analysis of transcription factors (Sp1 and TBP) binding affinities to the target sites to score the SNPs impact, based on “EMSA,” in silico prediction analysis, “sTRAP,” and RAVEN approaches

TFBS/Gene	Matrix ID	TF	Target site W/M	sTRAP^a^W/M	Affinity W/ M			EMSA p value/α	RAVEN^e^ score W/M
					EMSA	Prediction value ‐ln [KD]^d^	Ref.		
*COL1A1*	M00008 M00196 M00933 M00931M00932	Sp1	agggaaTG***G***GGGCGGGATGagggcct/ agggaaTG***T***GGGCGGGATGagggcct	6.70/5.85	0.095/0.039 (µM)^b^	—	Mann et al. (2001)	*p* < .001	TF not recognized for TFBS
*Hb ḉᴪ*	M00980 M00471	TBP	ctgccacacccaCATTAT***T***agaaaat/ctgccacaccCACATTAT***C***agaaaat	4.14/2.67	15.70/16.00^c^	17.72/18.28	Savinkova et al. (2013)	*α* < 10^–3^	TF not recognized for TFBS
*MBL2*	M00980 M00471	TBP	catctatttcTA***T***ATAgcctgcaccc/catctatttcTA***C***ATAgcctgcaccc	4.83/5.24, 2.59/0.98	17.39/16.66^c^	19.68/18.57	Savinkova et al. (2013)	*α* < 10^–3^	6.851(82.3%)/2.186 (73.1%)
*TF*	M00471 M00980	TBP	gccggcccTTTATAg***c***gcgcggggca/gccggcccTTTATAg***T***gcgcggggca	0.19/1.52 0.57/0.57	16.45/17.47^c^	18.91/19.43	Savinkova et al. (2013)	*α* < 10^–3^	TF not recognized for TFBS
*NOS2A*	M00980 M00471	TBP	atggggtgagTATAAATAc***t***tcttgg/atggggtgagTATAAATAc***C***tcttgg	0.09/0.09	20.14/20.25^c^	19.85/20.06	Savinkova et al. (2013)	*α* < 10^–3^	9.534 (87.7%)/11.614 (91.8%)
*FIX*	M00980 M00471	TBP	acagctcagcTT***G***TACTTTggtacaa/acagctcagcTT***C***TACTTTggtacaa	2.63/3.04, 0.33/0.33	14.49/14.51^c^	18.24/17.75	Savinkova et al. (2013)	*α* < 10^–3^	TF not recognized for TFBS

Abbreviations: M, Mutant; SNPs, single‐nucleotide polymorphisms; W, Wild Type.

a sTRAP model has applied different matrices to score the binding affinity of transcription factors (TFs), each of which applies diverse frames of transcription‐binding sites (TFBSs) for its computation. The score of the matrix with the highest energy in scanning TFBS consisting of target SNP has been presented. Excluding *MBL2*,* TF*, and *FIX*, for the others, different matrices gave the contradictory results.

b Sp1 results: The concentrations of radiolabeled competitor at 50% inhibition for "S" and "s" alleles, respectively; the lower concentration showed a higher binding affinity.

c TBP results: Equilibrium Dissociation Constant (−ln [K_D_]) which characterizes the binding affinity of TFs for TFBSs; the higher values showed the more affinities.

d −ln [K_D_] prediction value for TBP/TATA.

e Regulatory Analysis of Variation in Enhancers (RAVEN) is a Web‐based application utilized for detection and characterization of regulatory sequence variation.

One of TFBSs (in *Hb β* gene), in turn, corresponded with seven different mutant forms, due to the different contents and diverse SNPs. In the four out of six mutant TFBSs in the following genes, the binding sites could not be detected using RAVEN bioinformatics tool for SNP analysis (*COL1A1*,* Hb ḉᴪ*,* TF*,* FIX*). The results of RAVEN for other SNPs analysis were consistent with those produced by the experimental procedure. However, the data of 2 TFBSs out of 14 (inside *MBL2*, *NOS2A* genes) were contradictory to those produced by sTRAP. Concerning the TFBSs of *TF* and *FIX *genes, there were inconsistent scores in sTRAP analysis, when two different matrices were applied for binding affinity prediction of TBP for each of the target sites.

**Figure 1:**
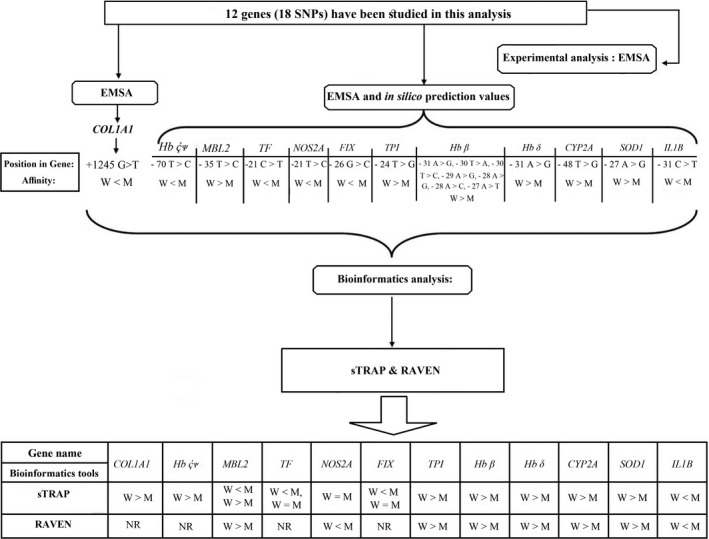
Workflow demonstrates the whole process in this study, consisting of experimental (EMSA) and bioinformatics data (sTRAP and RAVEN). W > M: The affinity is increased in the wild‐type sequences (W) versus mutant sequences (M). M > W: The affinity increased in mutant sequences (M) versus wild‐type sequences (W). W = M: There were no differences between two sequences. NR, not recognized

The experimental data, and in silico prediction values (Savinkova et al., 2013) for two certain TFs in interaction with their target TFBSs, versus those obtained from sTRAP and RAVEN in our study, are represented in Table 1. The whole details about the analysis process are categorized in Table S1. The workflow of the study also summarized in Figure 1.

## 4 DISCUSSION

The most of high‐throughput genomic data, with predictions based on SNPs variation, are still prone to error due to several reasons, including the bias mediated by the sequence context. In such cases, there would be a need to confirm the predicted results using orthogonal technology. Otherwise, the false positive and negative results would be inevitable, making the estimation of variants and their linkage to a disease, impractical (Cooper et al., 2018; Kamali et al., 2015).

However, this is not restricted to SNP predictions, the other estimations such as epigenetics, fusion proteins, and so on, would also be the cases of error profiling. As an example, the gold standard verification technology, for the high‐throughput data, next‐generation sequencing (NGS), is Sanger sequencing, specifically in the cases with quality scores <Q500 (Cooper et al., 2018; Park et al., 2014; Strom et al., 2014). Nonetheless, several other studies have substituted the alternative techniques including; targeted next‐generation sequencing, and mass spectrometric, for Sanger sequencing (Cooper et al., 2018; Sikkema‐Raddatz et al., 2013).

There are several bioinformatics datasets in the literature to predict the target sequences for the microRNAs, or vice versa (Kumar, Wong, Wong, Tizard, Moore, & Lefèvre, 2012; Piriyapongsa, Bootchai, Bootchai, Ngamphiw, & Tongsima, 2012; Agarwal, Bell, Nam, & Bartel, 2015); this is while some other databases have provided information on the binding microRNAs to the studied sequences, validated by experimental procedures. This might stand for the other example of bioinformatics data verification using experimental procedures (Huang et al., 2020; Karagkouni et al., 2018).

Although sTRAP, an online web tool constructed on the ChIP‐seq database, is fast and easily applied, in some cases, its results were paradoxical to the experimental data. We have looked at some TFs binding affinities by introducing SNPs inside their target‐binding sites, using sTRAP and RAVEN. Our results provided supporting evidence that at least in the case of Sp1 and TBP, sTRAP performance in six out of 18 SNPs was not consistent with those from documented experimental procedures (Mann et al., 2001; Savinkova et al., 2013). This might be indicative of sTRAP limitations in predicting the impacts of some of the SNPs in TFBSs and scoring the binding energies of their related TFs. The consequence of such restriction is to create some problems in quantifying the influence of the particular SNPs on human health and disease, estimating the functionalities of SNPs to waken or enhance the binding affinity of TFs, and testing related hypotheses based on SNPs variations in TFBSs.

Moreover, lack of any information on the probability (*p* value) between the binding energies of a TF for wild and mutant TFBSs, and also the absence of any cutoff of the significant differences makes the comparison between the two scores impractical. These features might imply the other restrictions of sTRAP performance. Such an explanation would also lead to the uncertainty in concluding that the results of the 12 out of 18 studied allelic variations exactly matched with the results of the experimental approaches. Nevertheless, there is a “log‐ratio ranking” of the affinities, which might not be properly responsive to the mentioned limitation, especially when the TF of the search is not among the highly ranked TFs.

The log ratio would rank the several TFs in a comparative model based on the higher absolute values of the difference between their binding affinities for the allelic variants of a specific motif. However, concerning an individual TF, the log ratio does not provide the probability value between two scores of the energy of TF for its binding sites (reference and mutant sequences). This finding of our study corresponded with the results of the other study (Macintyre, Bailey, Bailey, Haviv, & Kowalczyk, 2010). As an example, Skuse et al. (2014) used sTRAP to investigate if the sequence harboring rs237887 SNP, associated with social cognitive behavior, is a TF‐binding site and could induce altered gene expression. They reported the members of E26 transformation specific family of TFs being among those with significantly different binding affinities for their allelic motifs, ranked as top 11 candidate TFs. They established the hypothesis accordingly on the role of rs237887 SNP in the disease due to the altered TFs binding affinity, it makes. Their finding was according to the ranking of TFs based on the log ratio. However, as mentioned before, the relative logarithms do not provide a strong statistical tool to show the actual significant difference between the two values.

Furthermore, although the default threshold for the hit‐based method used by sTRAP is normally set on 5, the ranking of the TFs by the tool is mostly performed based on the threshold 0, being less stringent than 5. The outcome of this might be the highly ranked TFs with minimal affinity binding to the query motif, due to higher log ratio value only. The issue mentioned here was also experienced in our analysis, as we had to adjust the threshold on 0 to have a list of TFs in sTRAP output. So, the TFs with higher binding affinity for our motifs had lower scores of ranking as a result of the lower value of log ratio. This held true in most of the SNPs we analyzed.

In the view of the TFBSs inside *MBL2*,* TF*, and *FIX* genes in our analysis, there were discrepancies between the predicted scores, using mainly sTRAP, in the context of two different matrices applied for binding affinity prediction of TBP to any of the target sites (Table 1; Table S1). Such contradictory data might confuse the users to decide which result to consider. Besides, there is no *p* value between the two binding energies of a TF in such circumstances.

Manke et al. (2010) reported the investigation of 20 different SNPs in TFBSs, previously examined experimentally by Andersen et al. (2008), to evaluate the biophysical model, sTRAP, they had introduced. However, the Anderson et al. had examined a mix of either the nuclear extract, or cell extract, or the recombinant proteins as the sources of the putative TFs to analyze their binding energy.

Since cell and nuclear extract consist of a combination of several TFs, binding to the same motifs so, there might be a coincident activation of them. Therefore, the results of such an investigation might not indicate the binding energy of an individual target TF. Besides, this is not consistent with sTRAP, which exclusively computes the data of ChIP‐seq and applies the individual TFs (Thomas‐Chollier et al., 2011). We concluded that the protocol used by the authors to show the validity of sTRAP data might need to be designed more precisely. Nonetheless, extensive analysis of TFBSs as the cis‐acting elements are required to establish any corroborated correlation between the computational and experimental results. Although a biophysical model does not describe the biological systems 100% due to several parameters, there would be an absolute need for its approval; and validation by experimental procedures to find the level and degree of its discrepancies and deviations from the results of the wet‐lab experiments (Cooper et al., 2018). Without such challenges, sTRAP or any other biophysical model will not be known in terms of its performance. However, there might be a need, for extended analyses designed by several computational models and more integrated experimental data of SNPs analysis, for this purpose. Nevertheless, this is not a barrier to prevent researchers from looking at sTRAP in practice and experiment in a preliminary analysis. On the other side, sTRAP is one of the few accessible biophysical models to estimate the binding energy of the TFs for the wild and mutant sequences of the target TFBSs, web‐based, free of charge, able to produce numerical scores for the analysis, user‐friendly, specifically helping for the biologists with no need for strong background in mathematics and complex formulas, and no requirement for bioinformatics training to use such models. Although sTRAP has not been updated since its establishment in 2011, the named characterizations have made it the tool of choice among the existing computational models for many researchers in their ongoing projects to formulate the hypothesis to link the TFBSs alleles to the diseases by the prediction results (Cavalli et al., 2019; Huber et al., 2019; Skuse et al., 2014; Thormann et al., 2018). Such a frequent application of sTRAP makes its validation against experimental procedures valuable.

## 5 SUMMARY

The computational tool, sTRAP, in a user‐friendly manner, is capable to scan the TFBSs allele and predict the binding energy of TFs for their target sequences, simply using DNA sequence context. It is a practical biophysical tool which can be easily applied by even nonexperienced users.

Taken as a whole, sTRAP as a biophysical tool that takes advantage of multiple models requires being validated through experimental data and empirical measurements for assessing limitations and confidence. There would be a need to check the quality of the performance of the bioinformatics tool to accept the accuracy of its prediction (Cooper et al., 2018). So, a large scale of experimental data integrated with biophysical tool might be a prerequisite for sTRAP optimization and validations to precisely score the SNPs variations in TF‐TFBSs interactions. However, due to the complex scenario of TF activities in vivo (cross‐talking of TFs and coincident activation of them, cross‐talking of signal transduction pathways, numerous numbers of TFBS for an individual TF, existence of nonproductive interactions of genomic binding of TFs, chromatin modifications, cell type‐specific TFs, …; Adelaja & Hoffmann, 2019; Deplancke, Alpern, Alpern, & Gardeux, 2016; Huang et al., 2018; Keilwagen, Posch, Posch, & Grau, 2019; Mullen et al., 2011; Naidu, Kostov, Kostov, & Dinkova‐Kostova, 2015; Xin & Rohs, 2018), the results of neither EMSA nor sTRAP, analyzing individual TF‐binding energy could be an actual representation of the fate of nucleotide variations of TBFSs in vivo. Of note, the outcome of invalid estimations of a bioinformatics model might result in incorrect conclusions, and improper design of downstream experiments (Hayden, 2015). Our data indicated some limitations of sTRAP in the prediction of binding energy variations due to some SNPs inside TFBS in the human genome. To link an SNP to a disease or a phenotypic trait in a hypothesis, there might be a need to use sTRAP together with other bioinformatics models and validate their data by experimental sets.

Due to limitations in access to further experimental results in literature, this study has been presented as a preliminary analysis on the comparison of the experimental results and sTRAP data on the analysis of functional SNPs in the noncoding sequences of the human genome. However, for more comprehensive results, there would be a need to expand the study using several computational models and integrating more experimental data for analysis.

To the best of the authors’ knowledge, although several researchers have integrated “sTRAP” results in their studies and have compared them with the data obtained from other bioinformatics tools, this is the first report outlining the validation of the data of sTRAP by those of experimental approaches.

The data reported here add new information regarding sTRAP performance and might open a new window to the restrictions and capabilities of the biophysical tool, which requires being confirmed with an increased number of SNP analysis against the experimental sets.

## CONFLICT OF INTEREST

There was no conflict of interest to disclose.

## Supporting information

Table S1
